# Dyslipidemia Unmasked by the Treatment of Graves’ Disease: The Link Between Cholesterol Metabolism and Thyroid Hormones

**DOI:** 10.7759/cureus.89814

**Published:** 2025-08-11

**Authors:** Pranjali Sharma

**Affiliations:** 1 Endocrinology, Scripps Clinic, La Jolla, USA

**Keywords:** cholesterol, dyslipidemia, graves´disease, hypercholesterolemia, hyperlipidemia(hld), hyperthyroidism, hypothyroidism, ldl cholesterol, thyroid, thyroid dyslipidemia

## Abstract

Thyroid dysfunction can alter serum lipid levels, which can delay the diagnosis of hyperlipidemia. We report the case of a 44-year-old female in whom dyslipidemia was unmasked following the treatment of Graves’ disease. The diagnosis of Graves’ disease was made after the patient presented with fatigue, weight loss, palpitations, tremors, and shoulder pain. At that time, laboratory testing showed suppressed thyroid-stimulating hormone (TSH), free thyroxine (T4) of 2.39 ng/ml (normal: 0.7-1.48 ng/ml), total triiodothyronine (T3) of 3.36 ng/ml (normal: 0.4-1.93 ng/ml), thyroxine-receptor antibody (TRAb) of 9.07 IU/l (normal: ≤1.75 IU/l), thyroid-stimulating immunoglobulin (TSI) of 7.57 IU/l (normal: ≤0.54 IU/l) and a normal lipid panel (total cholesterol: 140 mg/dl, low-density lipoprotein (LDL) of 79 mg/dl, high-density lipoprotein (HDL) of 43 mg/dl, and triglyceride (TG) of 90 mg/dl). After treatment with methimazole for a year, the patient achieved euthyroidism (TSH: 2.043 mcIU/ml, free T4: 0.93 ng/ml, total T3: 1.28 ng/ml) but had dyslipidemia (total cholesterol: 257 mg/dl, LDL: 174 mg/dl, HDL: 51 mg/dl, TG: 161 mg/dl). There had been no change in the patient’s diet or lifestyle. It was thought that improvement in hyperthyroidism had unmasked underlying dyslipidemia.

The patient declined statin therapy and chose to pursue stricter lifestyle modifications. The cholesterol panel showed improvement eight months later (total cholesterol: 186 mg/dl, LDL: 114 mg/dl, HDL: 47 mg/dl, TG: 124 mg/dl) with ongoing euthyroidism (TSH: 2.099 mcIU/ml, free T4: 0.97 ng/ml, total T3: 1.24 ng/ml). This report highlights the close relationship between thyroid hormones and cholesterol metabolism. It reviews the pathways through which thyroid hormones affect cholesterol metabolism. The report emphasizes the importance of monitoring lipid profiles in patients with thyroid dysfunction. Conversely, thyroid function testing should be done in patients presenting with lipid abnormalities.

## Introduction

Cholesterol and triglycerides (TG) are water-insoluble lipids and require lipoproteins for transport. Based on size, composition, and type of apolipoprotein, there are seven different types of lipoproteins: chylomicrons, chylomicron remnants, very low-density lipoproteins (VLDL), low-density lipoproteins (LDL), intermediate-density lipoproteins (IDL), high-density lipoproteins (HDL), and lipoprotein a (LP(a)). Apolipoprotein B (ApoB) containing lipoproteins such as VLDL, LDL, chylomicrons, and LP(a) are pro-atherogenic, while HDL are anti-atherogenic. Lipids are produced from dietary and endogenous free fatty acids (FFA), and metabolized through the intestinal exogenous lipoprotein pathway and hepatic endogenous lipoprotein pathway [1].

The intestinal pathway is responsible for the transport of nutrients to the muscles and adipose tissue for energy production and storage through lipoprotein lipase (LPL). Dietary TG are hydrolysed to FFA, which form cholesterol esters in the intestinal lumen. Endogenous triglycerides, formed from dietary FFAs, and cholesterol esters are packaged into chylomicrons, which are released in the circulation through the lymphatic system [1]. 

The hepatic endogenous pathway is responsible for the production of VLDL and LDL to facilitate the transport of hepatic TG and cholesterol to peripheral tissue. FFAs enter hepatocytes via protein transporters such as fatty acid transporter proteins (FATP), liver fatty acid binding proteins (L-FATP), and fatty acid translocase (FAT). The addition of apolipoprotein B100 (Apo B100) via microsomal transfer protein (MTTP) leads to the production of VLDL. In the peripheral tissue, the TG in VLDL is hydrolysed to fatty acids, leading to IDL or VLDL remnants production. These particles are transported back to the liver, where hepatic LPL-mediated hydrolysis and addition of Apo B100 lead to the formation of LDL. LDL is then cleared by LDL receptors [1]. HDL particles, formed by cholesterol esters and apolipoprotein A1 (Apo A1), play an important role in reverse cholesterol transport between the peripheral tissue and the liver. HDL2 (anti-atherogenic) and HDL3 (atherogenic) are subparticles of HDL [2]. The cholesteryl ester transfer protein (CETP) mediates the transfer of cholesterol ester from HDL to ApoB-containing particles in exchange for TG, which are then metabolized by lipases [2]. The class B scavenger receptor B1 (SR-B1) promotes selective uptake of cholesterol from HDL in the liver. The HDL particle is then released into circulation to allow for further lipid exchange [1].

Thyroid hormones directly affect cholesterol metabolism through various transcriptional and non-transcriptional pathways. Within a normal thyroid-stimulating hormone (TSH) range, there is a linear relationship between total cholesterol, LDL, HDL, and TG levels and increasing TSH levels. Hypothyroidism is associated with an unfavorable effect on cholesterol metabolism, while hyperthyroidism tends to improve cholesterol profile [3]. Thyroid hormones also affect insulin resistance, oxidative stress, adipokine metabolism, endothelial metabolism, and body weight, which in turn affect cholesterol metabolism [3]. We present a case of a 44-year-old female with underlying dyslipidemia, who was thought to have a favorable change in cholesterol levels when she was diagnosed with Graves’ hyperthyroidism, and worsening dyslipidemia after undergoing treatment for Graves’ hyperthyroidism.

## Case presentation

A 44-year-old Caucasian female with no previous medical history or chronic medication intake was noted to have an elevated fasting LDL level (113 mg/dl, normal: <100 mg/dl) in September 2022 during her annual physical examination (Table 1). The remaining lipid panel was within normal range: total cholesterol: 181 mg/dl (normal: 7-200 mg/dl), HDL: 50 mg/dl (normal: >40 mg/dl), TG: 92 mg/dl (normal: 7-150 mg/dl) (Table 1). No specific causes for LDL elevation were identified. No specific recommendations were made regarding the management of elevated LDL levels.

**Table 1:** Trends in cholesterol and thyroid function testing LDL: low-density lipoprotein; HDL: high-density lipoprotein; TSH: thyroid-stimulating hormone; T4: thyroxine; T3: triiodothyronine; TRAb: TSH receptor antibody; TSI: thyroid-stimulating immunoglobulin

Variables	Normal	September 2022	October 2023	November 2024	March 2025	July 2025
Total cholesterol	7–200 mg/dl	181	140	257	220	186
LDL	<100 mg/dl	113	79	174	135	114
HDL	>40 mg/dl	50	43	51	52	47
Triglycerides	7–150 mg/dl	92	90	161	164	124
TSH	0.35–4.94 mcIU/ml	1.302	<0.008	2.043	1.965	2.099
Free T4	0.7–1.48 ng/dl	-	2.39	0.93	0.95	0.97
Total T3	0.4–1.93 ng/ml	-	3.36	1.28	1.14	1.24
Free T3	1.58–3.91 pg/ml	-	9.94	-	-	-
TRAb	≤1.75 IU/l	-	9.07	-	-	4.16
TSI	≤0.54 IU/l	-	7.57	-	-	2.93

A year later, in October 2023, the patient was diagnosed with hyperthyroidism due to Graves' disease when she presented with weight loss, fatigue, tremors, and shoulder pain. At this visit, her vitals were as follows: heart rate of 81/minute, respiratory rate of 14/minute, blood pressure of 101/65 mmHg, weight 122 lbs, and BMI of 21.61 kg/m^2^. Laboratory testing showed undetectable TSH, free thyroxine (T4) of 2.39 ng/ml (normal: 0.7-1.48 ng/ml), total triiodothyronine (T3) of 3.36 ng/ml (normal: 0.4-1.93 ng/ml), thyroxine-receptor antibody (TRAb) of 9.07 IU/l (normal: ≤1.75 IU/l), thyroid-stimulating immunoglobulin (TSI) of 7.57 IU/l (normal: ≤0.54 IU/l) and an improved lipid panel (total cholesterol: 140 mg/dl, LDL: 79 mg/dl, HDL: 43 mg/dl, TG: 90 mg/dl) (Table 1). The patient was advised that her lipid panel had improved. After discussion regarding hyperthyroidism therapy options such as anti-thyroid drug therapy, radioactive iodine ablation, and total thyroidectomy, the patient elected anti-thyroid drug therapy with methimazole 10 mg daily. Over the next several months, the patient showed clinical and biochemical improvement of Graves’ hyperthyroidism, and the methimazole dose was adjusted accordingly (Table 2).

**Table 2:** Thyroid function trends TSH: thyroid-stimulating hormone; T4: thyroxine; T3: triiodothyronine; TRAb: TSH receptor antibody; TSI: thyroid-stimulating immunoglobulin

Variables	Normal	September 2022	October 2023	January 2024	May 2024	August 2024	November 2024	March 2025	July 2025
TSH	0.35–4.94 mcIU/ml	1.302	<0.008	<0.008	1.129	3.097	2.043	1.965	2.099
Free T4	0.7–1.48 ng/dl	-	2.39	1.22	0.89	0.86	0.93	0.95	0.97
Total T3	0.4–1.93 ng/ml	-	3.36	1.29	1.31	1.22	1.28	1.14	1.24
Free T3	1.58–3.91 pg/ml	-	9.94	-	-	-	-	-	-
TRAb	≤1.75 IU/l	-	9.07	-	-	-	-	-	4.16
TSI	≤0.54 IU/l	-	7.57	-	-	-	-	-	2.93
Methimazole dose	Varying	Not applicable	10 mg daily	10 mg daily	10 mg daily	10 mg x 3 days, 5 mg x 4 days	10 mg x 2 days, 5 mg x 5 days	5 mg daily	5 mg daily

In November 2024, the patient underwent an annual physical examination, and fasting laboratory testing showed dyslipidemia (total cholesterol: 257 mg/dl, LDL: 174 mg/dl, HDL: 51 mg/dl, TG: 161 mg/dl) with normal thyroid function tests (TSH: 2.043 mcIU/ml, free T4: 0.93 ng/ml, total T3: 1.28 ng/ml) (Table 1). The patient did not report any major lifestyle changes, new medications, or supplements. Her weight was stable at 122 lbs with an unchanged BMI of 21.61 kg/m^2^. She was taking methimazole 10 mg daily five days a week and 5 mg two days a week. We hypothesized that the patient had underlying dyslipidemia, which was unmasked after treatment of hyperthyroidism.

The patient was advised to begin statin therapy, but she elected to make dietary and exercise modifications. She diligently followed a whole food plant-based and dairy-free diet and worked out at least five days a week. Fasting laboratory testing in March 2025 showed slight improvement in dyslipidemia (total cholesterol: 220 mg/dl, LDL: 135 mg/dl, HDL: 52 mg/dl, TG: 164 mg/dl) and continued euthyroidism (TSH: 1.965 mcIU/ml, free T4: 0.95 ng/ml, total T3: 1.14 ng/ml) (Table 1). While the patient was advised to consider statin therapy again, she elected to pursue more stringent lifestyle changes. Methimazole was decreased to 5 mg daily. As of July 2025, her cholesterol panel continues to show an improvement (total cholesterol: 186 mg/dl, LDL: 114 mg/dl, HDL: 47 mg/dl, TG: 124 mg/dl) with ongoing euthyroidism (TSH: 2.099 mcIU/ml, free: T4 0.97 ng/ml, total T3: 1.24 ng/ml). Since antibodies for Graves’ disease are still elevated (TRAb: 4.16 IU/l, TSI: 2.93 IU/l), we have elected to continue methimazole 5 mg daily for at least one more year.

## Discussion

This report describes a patient with underlying dyslipidemia that improved when she was diagnosed with Graves’ hyperthyroidism and then deteriorated after Graves’ hyperthyroidism was optimally managed. It highlights the close relationship between cholesterol and thyroid hormone levels. Thyroid hormones regulate the cholesterol pathway through transcriptional regulation via the thyroid hormone receptors (THR) and non-transcriptional effects. 

Transcriptional pathways

The enzyme involved in the first step of cholesterol synthesis, 3-hydroxy-3-methylglutaryl-coenzyme A (HMG-CoA) reductase, is induced by thyroid hormones [1]. Both lipogenesis and lipolysis are regulated by thyroid hormones. Thyroid hormones stimulate the transcription of genes involved in FFA lipogenesis, such as fatty acid synthase [4]. They also play a role in the expression and activities of transcription factors such as the sterol regulatory element binding protein 1C, carbohydrate-responsive element binding protein, which are involved in hepatic lipogenesis [4]. Thyroid hormones stimulate lipolysis in white adipose tissue, which generates FFAs. Theoretically, under euthyroid conditions, TG production should increase with FFA production, but this does not occur due to thyroid hormone-mediated lipolysis and further FFA metabolism [4]. 

The process of thyroid hormone-mediated lipolysis involves mobilization, degradation, and β-oxidation of FFAs in the liver. This is mediated by hepatic lipase, lipophagy, and mitochondrial oxidation of fatty acids. The expression and activity of hepatic lipase are dependent on thyroid hormones [4]. Thyroid hormones increase the number of autophagosomes and lysosomes and activate transcription factors and proteins involved in the process of lipophagy. At the mitochondrial level, thyroid hormones control the oxidation of fatty acids by regulation of transcription factors such as PPAR g co-activator 1a and enzymes such as the carnitine O-palmitoyltransferase 1 and medium chain acyl-CoA dehydrogenase [4]. T3 also upregulates apolipoprotein AV (ApoAV), which helps with TG regulation [2]. The FFA protein transporters (FATP, FAT) are regulated by thyroid hormones in a tissue-specific manner. In hyperthyroidism, fatty acid uptake is increased in the liver and muscles, whereas in hypothyroidism, fatty acid uptake is increased in the white adipose tissue and decreased in the liver due to decreased expression of hepatic FAT and FABP [4]. TG levels can be normal or decreased in hyperthyroidism, while they are normal or increased in hypothyroidism.

T3 binds to specific thyroid hormone-responsive elements (TREs), which activate the LDL receptor gene and upregulate LDL receptors [3]. LDL receptor activation by thyroid hormone increases the fractional catabolic rate of ApoB100, which is required for LDL formation. It has also been reported that thyroid hormones regulate cholesterol levels through microRNAs in a non-TRE-mediated manner. Thyroid hormone-mediated expression of miR181d decreases the transcription factor for sterol O-acyltransferase 2, the enzyme required to convert cholesterol to cholesterol esters. This decreases LDL production [4]. Additionally, T4 binds to LDL cholesterol to form a T4-LDL complex, which is recognized by the LDL receptor, allowing for LDL uptake into the cell [2]. Upregulated LDL receptors increase LDL clearance. Thyroid hormones also increase transcription of LDL receptor-related protein 1, which removes chylomicron remnants and VLDL [4]. Peripheral lipoprotein lipase is also regulated by thyroid hormones [2]. Thus, thyroid hormones affect both LDL synthesis and degradation in the liver and peripheral tissues [2]. In hypothyroidism, there is an increase in LDL levels due to overall decreased degradation of LDL, while hyperthyroidism is associated with lower LDL levels due to increased degradation of LDL.

Thyroid hormones play a role in HDL metabolism through various mechanisms. Thyroid hormones induce the gene and protein expression of Apo A1 required in the production of HDL, and SR-B1, needed for hepatic uptake of cholesterol from HDL [4]. Thyroid hormones also increase CETP activity, which aids the exchange of cholesteryl esters between HDL, VLDL, and LDL [4]. CETP transports TG to HDL2, which is then hydrolyzed by hepatic lipases. Hepatic lipase expression and activity are dependent on the status of thyroid function [4]. Hypothyroidism is associated with decreased CETP and hepatic lipase activity, which does not allow hydrolysis of HDL, leading to an increase in HDL levels. Since CETP and hepatic lipase activity are increased in hyperthyroidism, HDL levels are lower. The adipose triglyceride lipase (ATGL) is also present in hepatic cells. Thyroid hormones increase the recruitment of ATGL, thereby facilitating lipolysis [4].

Non-transcriptional pathways

Thyroid hormones also exert biological actions that do not require THRs. T3 regulates hepatic lipogenesis through the PI3K-RACa and the cAMP-protein kinase A pathways. Thyroid hormone derivative 3,5-diiodothyronine has been shown to increase hepatic fatty acid oxidation, activate hepatic lipase, and suppress hepatic lipogenesis, thereby decreasing LDL levels [4]. While rodent studies have suggested that TSH also regulates cholesterol synthesis, it has been difficult to assess the action of TSH independent of the action of thyroid hormones [4]. 

Thus, based on the mechanisms above, hyperthyroidism is associated with decreased total cholesterol, decreased LDL cholesterol, normal or decreased HDL cholesterol, and normal or decreased TG levels, primarily driven by increased LDL turnover and increased FFA metabolism. Hypothyroidism, on the other hand, is characterized by elevated total cholesterol, increased LDL, normal or increased HDL cholesterol, and normal or increased TG levels, due to decreased cholesterol excretion, decreased LDL turnover, and increased ApoB levels [2]. 

Our patient had dyslipidemia onset in 2022 when her LDL level was 113 mg/dl; however, this was not addressed due to an otherwise normal cholesterol panel. When she was diagnosed with hyperthyroidism, her cholesterol levels were deemed well-controlled. Retrospectively, we believe that elevated thyroid hormones led to lower cholesterol levels through mechanisms described above. Once hyperthyroidism was optimally treated, decreased LDL turnover and FFA metabolism led to higher cholesterol, LDL, HDL, and TG levels, causing dyslipidemia. The changes in the cholesterol levels in our patient are consistent with thyroid-related lipid changes reported in the literature. 

The management of the underlying thyroid problem is important when dealing with cholesterol issues. While treatment of hypothyroidism involves thyroid hormone supplementation, hyperthyroidism may be treated through anti-thyroid drug therapy, radioactive iodine ablation, or thyroidectomy [5,6]. Our patient opted for anti-thyroid drug therapy with methimazole after reviewing her options, and she responded well to the medication. 

The management of dyslipidemia involves the assessment of cardiovascular risk, intensive counseling regarding diet and physical activity, and consideration of medical therapy with statins. Additional medications such as ezetimibe, bempedoic acid, proprotein convertase subtilisin/kexin type 9 (PCSK-9) inhibitors, inclisiran, or eicosapentaenoic acid (EPA) may be used for additional cholesterol lowering [7]. Our patient had low cardiovascular risk. She was deemed a candidate for medical therapy due to the degree of dyslipidemia, but she opted to pursue strict lifestyle changes instead, which led to significant improvement in her condition. 

## Conclusions

Thyroid hormones affect cholesterol levels via both transcriptional and non-transcriptional mechanisms. Thyroid hormones regulate the synthesis and clearance of all lipoproteins, including LDL, HDL, VLDL, and TG. While hypothyroidism can worsen cholesterol levels, hyperthyroidism decreases cholesterol levels and may mask underlying dyslipidemia. It is important to monitor cholesterol levels when treating thyroid dysfunction. Conversely, it is important to rule out a thyroid issue when abnormally low or high cholesterol levels are noted. Management of thyroid dysfunction-related dyslipidemia involves appropriate treatment of the underlying thyroid issue and treatment of dyslipidemia through lifestyle changes, and when needed, medication.
